# Radiation Oncology in the 21st Century: Prospective Randomized Trials That Changed Practice… or Didn’t!

**DOI:** 10.3389/fonc.2018.00130

**Published:** 2018-04-30

**Authors:** Kaveh Zakeri, C. Norman Coleman, Bhadrasain Vikram

**Affiliations:** ^1^Radiation Research Program, Division of Cancer Treatment and Diagnosis, National Cancer Institute, Rockville, MD, United States; ^2^Department of Radiation Medicine and Applied Sciences, University of California San Diego, La Jolla, CA, United States

**Keywords:** randomized trials, radiation oncology, survival, local control, adverse events

## Abstract

In a two-part article published in 2009, we discussed the limitations of conventional radiation therapy, the challenges of studying new technologies in radiation oncology, and summarized the state-of-the science for various malignancies (1, 2). Here, we summarize some of the most important prospective, randomized trials that during the intervening years have attempted to improve the tumor control and/or decrease the adverse effects of radiation therapy. For consistency, we have focused here on the null and alternate hypotheses as articulated by the investigators at the onset of each trial, since the outcome of the investigational treatment should be considered clinically significant only if the null hypothesis was rejected. The readers (and patients) are of course free to make their own judgments about the clinical significance of the results when the null hypothesis was not rejected.

## Introduction

An overview of recent randomized trials in radiation oncology provides a useful primer on the current state of the field for students, physicians, and researchers. Summarizing the current survival, tumor control, and toxicity data for various disease sites using the highest level of evidence can guide future efforts to improve treatment outcomes for cancer patients. In this article, we included all prospective randomized trials involving radiation therapy that are required to understand the current scope of radiation oncology practice. Many of these trials have influenced clinical practice by either establishing a new standard of care or helping resolve an important question in the management of patients. The article discusses both publically and privately funded clinical trials from across the globe.

### Glioblastoma

Stupp et al. (3) randomized 695 patients to receive tumor-treating fields (TTFs) or not after the completion of chemoradiotherapy. TTFs are an antimitotic treatment that disrupt cellular division using alternating electric fields applied to a patient’s scalp. The null hypothesis was that adding TTF did not prolong progression-free survival (PFS). The null hypothesis was rejected because, at the first interim analysis, the median PFS was 7.1 months with TTF and 4.0 months without [hazard ratio (HR) = 0.62; *p* = 0.001]. Median overall survival (OS) was also 4.9 months longer after TTF (HR = 0.64; *p* = 0.004), but even with TTF, one-half of the patients died within 20.5 months, so there is ample room for further improvement.

### Anaplastic Glioma and Oligodendroglioma

Cairncross et al. (4) randomized 291 patients with anaplastic oligodendrogliomas and oligoastrocytomas to receive procarbazine, lomustine, and vincristine (PCV) plus radiotherapy or radiotherapy alone after resection. The null hypothesis was that OS was not prolonged by PCV. The null hypothesis could not be rejected because median OS was similar between the two arms (4.6 vs 4.7 years). Secondary analyses, however, showed that, with or without PCV, the 126 patients with 1p19q codeleted tumors lived much longer than those with non-codeleted tumors, and only among those with codeleted tumors, PCV markedly prolonged the median OS compared to radiotherapy alone (14.7 vs 7.3 years; HR = 0.59; *p* = 0.03).

Van den Bent et al. (5) conducted a randomized trial of PCV plus radiation vs radiation alone following resection in 368 patients with anaplastic oligodendrogliomas. The null hypothesis was that PCV did not prolong OS by 12 months or longer. After a median follow-up of 140 months, the addition of PCV did prolong median OS by 11.7 months (42.3 vs 30.6 months, HR = 0.75; *p* = 0.018), but since this was a secondary analysis, the null hypothesis could not be formally rejected. Eighty patients had 1p19q codeleted tumors and similar to Cairncross et al. (4), the OS of these patients tended to be longer, especially if they had received PCV.

In the CATNON trial (6), patients with newly diagnosed 1p/19q non-codeleted anaplastic gliomas were randomized in 2 × 2 factorial design to radiotherapy (59.4 Gy in 33 fractions) alone or with concurrent temozolomide, and with or without adjuvant temozolomide. The null hypothesis was that, compared with radiation alone, adjuvant temozolomide did not prolong OS. The null hypothesis was rejected because an interim analysis after enrolling 745 patients showed longer OS with adjuvant temozolomide (5-year OS 55.9 vs 44.1%; HR = 0.65; *p* = 0.0014). This trial continues for evaluating the benefit of concurrent temozolomide.

### Low-Grade Glioma

Buckner et al. (7) conducted a randomized trial in 251 patients with grade 2 astrocytoma, oligoastrocytoma, or oligodendroglioma. The patients were either younger than 40 years and had a subtotal resection or biopsy, or were older than 40 and had a biopsy or resection of any of the tumor; very few patients had total resection. The patients were randomized to radiation alone or followed by PCV. The null hypothesis was that the addition of PCV did not improve the 5-year OS by 21% (corresponding to HR of 0.46). The null hypothesis could not be rejected because, after a median follow-up of 5.9 years, the 5-year OS after radiation plus PCV was non-significantly 9% better (72 vs 63% for radiation alone; HR = 0.72; *p* = 0.33). An exploratory analysis, after a median follow-up of 11.9 years, however suggested that patients receiving PCV lived much longer (median OS 13.3 vs 7.8 years; HR = 0.59; *p* = 0.003). Additional exploratory analyses suggested that those with oligodendriogliomas lived the longest, especially if they had received PCV, as did those whose tumors exhibited the R132H mutation in the isocitrate dehydrogenase 1 gene (IDH1 R132H mutation).

### Brain Metastases

Brown et al. (8) conducted a randomized trial to compare stereotactic radiosurgery (SRS) alone vs whole brain radiation therapy (WBRT) plus SRS in 213 patients with 1–3 brain metastases; most had lung cancer and about 50% had a single metastasis. The null hypothesis was that cognitive deterioration at 3 months would not be less after SRS alone. The null hypothesis was rejected because the observed rate of cognitive deterioration at 3 months was 63.5% after SRS alone compared to 91.7% after WBRT plus SRS (*p* < 0.001). Intracranial failure, however, occurred much sooner after SRS alone (HR = 3.6; *p* < 0.001). After SRS, one-half of the patients died within 10.4 months; therefore, there remains ample room for improvement.

Another trial (9) randomized 194 patients who had undergone resection of a brain metastasis to SRS vs WBRT. Eligible patients had a surgical cavity measuring less than 5.0 cm in maximal diameter and could have up to three unresected metastases. This study had co-primary endpoints of cognitive-deterioration-free survival and OS. The trial was powered to detect a 20% benefit in cognitive-deterioration-free survival at 6 months after randomization, and a 2-month improvement in median OS with SRS. Cognitive deterioration at 6 months was observed in 52% of the patients after SRS compared to 85% after WBRT (*p* < 0.00031); therefore, that null hypothesis was rejected. There was, however, no improvement in median OS with SRS (12.2 vs 11.6 months; *p* = 0.70) and, furthermore, intracranial failure occurred sooner after SRS than WBRT (median 6.4 vs 27.5 months; *p* < 0.0001). It is sobering that the OS of such surgery-eligible patients has remained essentially unchanged since the landmark 1990 Patchell trial (10).

### Head and Neck Cancers

A 94-patient randomized clinical trial (11) compared intensity-modulated radiation therapy (IMRT) vs 3D conformal radiation (3DCRT) therapy for tumors of the oropharynx and hypopharynx. The null hypothesis was that grade 2 or worse xerostomia 12 months later was not better after IMRT. The null hypothesis was rejected because such xerostomia was observed in 38% of the patients in the IMRT arm vs 74% in the 3DCRT arm (*p* = 0.0027). The trial was not powered to reliably assess small differences in locoregional PFS or OS. Figure 3 in Nutting et al. raised concerns about possibly worse long-term tumor control in the IMRT arm and the authors stated: “Long-term follow-up of patients is ongoing.” However, that has not yet been published 6 years after the original publication.

### Non-Small Cell Lung Cancer (NSCLC)

#### Early Stage NSCLC

Nyman et al. (12) randomized 102 patients with medically inoperable stage I NSCLC to stereotactic body radiation therapy (SBRT) (66 Gy in 3 fractions) or conventional irradiation (70 Gy in 35 fractions). The null hypothesis was that the 3-year PFS was not superior after SBRT. The null hypothesis could not be rejected because after a median follow-up of 37 months, the 3-year PFS was 42% in each arm. Secondary analyses revealed less toxicity after SBRT, however, than after conventional irradiation.

Videtic et al. (13) randomized 94 patients with medically inoperable stage I peripheral NSCLC to either 48 Gy in four fractions or 34 Gy in a single fraction. The null hypothesis was that each regimen would have an unacceptable adverse event rate of 17% (grade 3 or worse). The rate of grade 3 or worse adverse events was only 10.3% in the single fraction arm and 13.3% in the 4-fraction arm. Furthermore, the local control rate after 1 year was 97% in the single-fraction arm and 92.7% in the 4-fraction arm. The combination of low toxicity and high tumor control with greater convenience makes the single-fraction option very attractive for further development.

Two prospective randomized trials of SBRT vs lobectomy were started. The STARS trial had a primary endpoint of OS and the ROSEL trial had a primary endpoint of local and regional control. The trials both closed prematurely due to slow accrual. An exploratory pooled analysis of data from both trials (14) included 58 patients and suggested that 3-year OS after SBRT was superior (95 vs 79% after surgery; *p* = 0.037).

#### Locally Advanced NSCLC

A randomized trial (15) in 465 patients with locally advanced NSCLC undergoing concomitant chemoradiotherapy tested whether 74 Gy improved OS compared with 60 Gy; unexpectedly, it found that OS was inferior after 74 Gy (median 20.3 vs 28.7 months for the 60 Gy arm; *p* = 0.008). Median OS with or without cetuximab was similar (25.0 vs 24.0 months) in this same trial that employed a 2 × 2 factorial design. An analysis of dose to cardiac structures revealed numerous cardiac dose volumes including mean pericardium dose were correlated with worse survival, and mean pericardium dose was also associated with grade 3 or higher pneumonitis (16).

Liao et al. (17) randomized 149 patients to receive intensity-modulated photon therapy or 3D-proton therapy. The primary endpoint was treatment failure (TF) rate, a composite of local failure and/or grade 3, or worse pneumonitis. The null hypothesis was that the TF rate at 12 months was not better after proton therapy. The null hypothesis could not be rejected because the 12-month TF rate was non-significantly worse after proton therapy (21.1 vs 17.4% after photons). The mean cardiac dose was lower in the proton arm (*p* = 0.002) but that did not translate into improved outcomes.

Antonia et al. randomized 713 patients to receive the anti-PDL1 antibody durvalumab or placebo after concurrent chemoradiation (18). The trial had co-primary endpoints of OS and PFS, and it was powered to detect a HR of 0.67 for PFS and a HR of 0.73 for OS. The median PFS was 16.8 months with durvalumab and 5.6 months with placebo (HR = 0.52; *p* < 0.001); therefore, that null hypothesis (that PFS after durvalumab was not superior to placebo) was rejected. It is too early to determine if OS is prolonged by durvalumab or not and the trial is continuing.

### Small Cell Lung Cancer (SCLC)

#### Limited Disease (LD)

Faivre-Finn et al. (19) randomized 547 patients with LD-SCLC to chemoradiation with 45 Gy in 30 fractions (given twice a day) or 66 Gy in 33 fractions (given once a day). The null hypothesis was that the 2-year OS was not superior after 66 Gy. The null hypothesis could not be rejected because 2-year OS was not significantly different (51% after 66 Gy vs 56% after 45 Gy; *p* = 0.14). The authors suggested that the standard of care should, therefore, remain 45 Gy in 30 fractions (given twice a day).

#### Extensive Disease (ED)

Slotman et al. (20) randomized 498 patients who had responded to chemotherapy to receive or not receive thoracic radiotherapy to 30 Gy in 10 fractions. The null hypothesis was that thoracic radiotherapy did not improve the 1-year OS by 10% (to 37 from 27%; HR = 0.76). The null hypothesis could not be rejected because, with a median follow-up of 24 months, the 1-year OS after thoracic radiotherapy was not significantly different (33% with thoracic radiotherapy vs 28% without; HR = 0.84; *p* = 0.066). In secondary analyses, the median OS was the same in both groups (8 months), but the 2-year OS was significantly longer with thoracic radiotherapy (13 vs 3%; *p* = 0.004).

Consolidative radiotherapy (to the chest and to any extracranial metastases that did not show a complete response after chemotherapy) was evaluated in a 97-patient randomized trial (21). The null hypothesis was that adding radiotherapy did not reduce the risk of death. The null hypothesis could not be rejected and the trial was terminated early for futility because a planned interim analysis demonstrated numerically but not significantly worse 1-year OS after consolidative radiotherapy (50.8 vs 60.1% without radiotherapy; *p* = 0.21).

#### Prophylactic Cranial Irradiation (PCI)

Takahashi et al. (22) conducted a 224-patient randomized trial of PCI vs observation for patients with ED SCLC who had no brain metastases on magnetic resonance imaging (MRI). The null hypothesis was that PCI did not improve OS. The null hypothesis could not be rejected and the trial was closed early for futility when a planned interim analysis revealed non-significantly worse median OS in the PCI arm (11.6 vs 13.7 months in the observation arm; HR = 1.27; *p* = 0.094).

### Breast Cancer

In a prospective randomized trial, 4,004 women (with centrally or medially located breast tumors, or laterally located tumors with axillary involvement) received chest wall irradiation with or without regional nodal irradiation (23). The null hypothesis was that regional nodal irradiation did not improve the 10-year OS by 4% (to 79 from 75%). The null hypothesis could not be rejected because, after a median follow-up of 10.9 years, OS was non-significantly improved by 1.6% in the nodal irradiation group (82.3 vs 80.7%; *p* = 0.06).

A similar randomized trial was conducted in 1,832 women with node-positive or high-risk node-negative breast cancer (24). The null hypothesis was that regional nodal irradiation did not improve the 5-year OS by 5% (to 85 from 80%). The null hypothesis could not be rejected because, after a median follow-up of 9.5 years, OS was non-significantly better by only 1% in the nodal irradiation group (82.8 vs 81.8%; *p* = 0.38).

These two trials suggested that with regard to OS regional nodal radiation was not superior to treating without it.

#### Accelerated Partial Breast Radiotherapy

Intraoperative radiation therapy (IORT) following lumpectomy for early breast cancer can spare many patients the need for “standard” whole breast radiation therapy. A 3,451-patient randomized trial compared IORT vs whole breast radiotherapy (25). The null hypothesis was that IORT would lead to worse local control in the irradiated breast (2.5% more local recurrences within 5 years). This null hypothesis was not rejected; local recurrences were more common after IORT (3.3 vs 1.3%; *p* = 0.042), and the 95% confidence interval (CI) included the pre-specified non-inferiority margin (26).

A similar trial randomized 1,305 women to IORT vs whole breast radiotherapy (27). The null hypothesis was that IORT would lead to worse local control in the irradiated breast (4.5% more local recurrences within 5 years). After median follow-up of 5.8 years, the null hypothesis was rejected. Although there were significantly more local recurrences after IORT (4.4%, 95% CI 2.7–6.1 vs 0.4%, 95% CI 0.0–1.0%; *p* < 0.0001), the difference was within the pre-specified equivalence margin.

Another randomized trial compared whole breast radiotherapy to interstitial partial breast brachytherapy in 1,184 patients (28). The null hypothesis was that brachytherapy would lead to worse local control in the irradiated breast (3% more local recurrences within 5 years). The null hypothesis was rejected because local recurrences occurred 0.52% (95% CI −0.72 to 1.75) more after brachytherapy (1.44 vs 0.92%), which was within the pre-specified non-inferiority margin.

#### Hypofractionated Whole Breast Radiotherapy

The START-A trial (29) randomized 2,236 women with completely excised invasive breast cancer (pT1-3a, pN0-1, M0) to conventional (50 Gy in 25 fractions) vs hypofractionated (41.6 Gy in 13 fractions, or 39 Gy in 13 fractions) radiotherapy. The null hypothesis was that, after hypofractionation, the local control would be worse (5% more local recurrences within 5 years). After a median follow-up of 5.1 years, the null hypothesis was rejected for both comparisons. The 41.6 Gy arm, 50 Gy arm, and 39 Gy arm had local recurrences of 3.5, 3.6, and 5.2%. The absolute difference in local control at 5 years between the 50 and 41.6 Gy was 0.2% (95% CI: −1.3 to 2.6%) and the difference between the 50 and 39 Gy was 0.9% (95% CI: −0.8 to 3.7%). The 95% CI for the difference excludes the pre-specified non-inferiority margin of 5%.

The START B trial (30) similarly randomized 2,215 women to conventional (50 Gy in 25 fractions) vs hypofractionated (40 Gy in 15 fractions) radiotherapy. The null hypothesis was that, after hypofractionation, the local control would be worse (5% more local recurrences within 5 years). The null hypothesis was rejected since a 5% difference in local recurrences was excluded. The rate of local recurrences at 5 years after 40 Gy in 15 fractions was 2.2% compared to 3.3% after 50 Gy in 25 fractions (absolute difference −0.7%; 95% CI: −1.7 to 0.9%).

Longer term results of these two trials (31) confirmed not only that the tumor control was non-inferior after hypofractionated radiotherapy but also that adverse effects were significantly less common after 40 Gy in 15 fractions or 39 Gy in 13 fractions than after 50 Gy in 25 fractions.

Another 1,234 patient randomized trial compared a conventional (50 Gy in 25 fractions) schedule to a hypofractionated (42.5 Gy in 16 fractions) schedule after breast-conserving surgery (32). The null hypothesis was that hypofractionated radiotherapy would result in 5% more local recurrences. The null hypothesis was rejected after a median follow-up of 12 years. The hypofractionated radiotherapy arm had a local recurrence rate of 6.2 vs 6.7% for conventional fractionation (*p* < 0.001 in favor of non-inferiority).

### Esophageal Cancer

Van Hagen et al. (33) randomized 368 patients with resectable esophageal or esophagogastric-junction tumors to undergo surgery alone or neoadjuvant chemoradiation followed by surgery. The null hypothesis was that adding neoadjuvant therapy did not improve OS by 6 months (to 22 from 16 months). The null hypothesis was rejected because the median OS improved by 21.4 months (to 49.4 from 24.0 months after surgery alone; HR 0.657; *p* = 0.003).

### Pancreas Cancer

Hammel et al. (34) randomized 449 patients with locally advanced pancreas cancer to chemotherapy vs chemotherapy plus radiotherapy. The null hypothesis was that adding radiotherapy did not increase OS. The null hypothesis could not be rejected because median OS was not significantly different (15.2 months for chemoradiotherapy vs 16.5 months for chemotherapy; *p* = 0.83). The same trial, using a 2 × 2 factorial design, also found that adding erlotinib to gemcitabine did not prolong median OS.

### Prostate Cancer

#### Early Prostate Cancer

Hamdy et al. (35) randomized 1,643 men with clinically localized prostate cancer to active monitoring, surgery, or radiotherapy [from 1999 to 2009, a total of 82,429 men aged 50–69 years in the United Kingdom had a prostate-specific antigen (PSA) test; 2,664 received a diagnosis of localized prostate cancer and 1,643 agreed to undergo randomization to active monitoring, radical prostatectomy, or radiotherapy]. The null hypothesis was that prostate-cancer mortality after 10 years of follow-up was not different between the active monitoring and treatment arms. The null hypothesis could not be rejected because, after a median follow-up of 10 years, there was no significant difference among the three arms in the number of deaths from prostate cancer (8 after active monitoring, 5 after surgery, and 4 after radiotherapy; *p* = 0.48). Furthermore, the OS was also similar among the three arms (*p* = 0.87).

Michalski et al. (36) randomized 1,532 men with localized prostate cancer to receive 79.2 or 70.2 Gy of radiotherapy (eligible patients had clinical stage T1b-T2b and Gleason Score 2–6 and PSA 10–20, or clinical stage T1b-T2b and Gleason Score 7 and PSA < 15). The null hypothesis was that giving the higher dose did not improve the OS. The null hypothesis could not be rejected because, after a median follow-up of 8.4 years, the 5-year OS was nearly identical in the two arms (88 vs 89%) while the 8-year OS rate was also similar (76% in the 79.2 Gy arm and 75% in the 70.2 Gy arm). The patients receiving 79.2 Gy, however, suffered significantly more late grade 2 or worse gastrointestinal and genitourinary toxicity than those receiving 70.2 Gy.

#### Hypofractionated Prostate Radiotherapy

The CHHiP trial (37) randomized 3,216 men to receive 74 Gy in 37 fractions, 60 Gy in 20 fractions, or 57 Gy in 19 fractions. The null hypothesis was that biochemical or clinical failures were 5% more likely after hypofractionation than 74 Gy in 37 fractions. After 5 years, the null hypothesis was rejected for the 20-fraction arm but not for the 19-fraction arm. Fewer failures were observed in the 20-fraction arm (9.4 vs 11.7% after 34 fractions; *p* = 0.0018 in favor of non-inferiority). The 19-fraction arm had more failures than the 37-fraction arm (14.1 vs 11.7%; *p* = 0.48 not in favor of non-inferiority).

Lee et al. (38) randomized 1,092 men to receive 73.8 Gy in 41 fractions or 70 Gy in 28 fractions. The null hypothesis was that the 5-year disease-free survival (DFS) was worse in the 28-fraction arm. The null hypothesis was rejected because, after a median follow-up of 5.8 years, the 5-year DFS non-inferiority criterion was met (86.3% with 28 fractions vs 85.3% with 41 fractions; *p* < 0.001 in favor of non-inferiority).

Catton et al. (39) randomized 1,206 men with intermediate-risk prostate cancer to receive 78 Gy in 39 fractions or 60 Gy in 20 fractions. The null hypothesis was that biochemical–clinical failures would be more frequent in the 20-fraction arm. The null hypothesis was rejected because, after a median follow-up of 6.0 years, the 5-year biochemical–clinical failure rate was identical in the two arms (15%).

#### Locally Advanced or Metastatic Prostate Cancer

Abiraterone acetate is a selective, irreversible inhibitor of CYP17, an enzyme that is critical in the production of androgens in the testes, adrenal glands, and prostate-tumor tissue. Inhibition of CYP17 in combination with androgen deprivation therapy (ADT) results in a more effective androgen depletion than can be induced by surgical castration or by GnRH analogs alone. James et al. (40) randomized 1,917 patients with locally advanced or metastatic prostate cancer to ADT alone or ADT plus abiraterone. Local radiotherapy was mandated for patients with node-negative, non-metastatic disease (*N* = 537) and encouraged for node-positive disease. Altogether, 786 patients received pelvic radiotherapy. The null hypothesis was that adding abiraterone did not improve the OS. The null hypothesis was rejected because, after a median follow-up of 40 months, there were far fewer deaths in the abiraterone arm (184 vs 262, HR = 0.63; *p* < 0.001). Subset analysis of the non-metastatic group is awaited.

#### Metastatic Prostate Cancer

Parker et al. (41) randomized 921 men with castrate-resistant prostate cancer and bone metastases to radium-223 or placebo. The null hypothesis was that radium-223 did not prolong OS with an HR of 0.76. The null hypothesis was rejected because OS was much longer after radium-223 (14.9 vs 11.3 months; HR = 0.70; *p* < 0.001).

### Bladder Cancer

James et al. (42) randomized 360 patients with muscle-invasive bladder cancer to radiotherapy with or without concurrent chemotherapy (mitomycin C plus 5-fluorouracil). The null hypothesis was that adding chemotherapy did not improve the locoregional DFS by 15% (to 65 from 50%; HR = 0.62). The null hypothesis was rejected because, after a median follow-up of 69.9 months, the 2-year locoregional DFS was significantly increased (to 67 from 54%; HR = 0.68; *p* = 0.03). Five-year rate of OS was 48% in the chemoradiotherapy arm and 35% in the radiotherapy arm (HR = 0.82; *p* = 0.16). Grade 3 or 4 adverse events were more common in the chemoradiotherapy group.

In this same trial, employing a 2 × 2 factorial design, 219 patients also agreed to be randomized between whole bladder vs partial bladder boost (43). The null hypothesis was that partial bladder boost was 10% worse regarding the 2-year locoregional control rate. This null hypothesis, however, could not be rejected. The 2-year locoregional control rate was worse by 6.4% after partial bladder boost, and the 95% CI included the pre-specified non-inferiority margin. With regard to grade 3/4 toxicity, there was no observable difference between partial bladder and whole bladder boost.

### Gynecologic Cancer

Klopp et al. (44) randomized 289 patients with cervical or endometrial cancer after definitive surgery to conventional radiotherapy vs IMRT. The primary endpoint was a decline on the bowel domain of the Expanded Prostate Cancer Index Composite (EPIC) scale. The null hypothesis was that acute gastrointestinal toxicity as measured by the decline at 5 weeks would not be less after IMRT. That null hypothesis was rejected because patients treated with IMRT had a significantly lower decline in EPIC bowel scores at 5 weeks when compared to conventional radiation (18.6 vs 23.6, *p* = 0.048). No mention was made by the authors of the null hypothesis regarding tumor control; that information is awaited to feel confident that the tumor control was not compromised.

### Rectal Cancer

Ngan et al. (45) randomized 326 patients with T3 rectal cancer within 12 cm of the anal verge to either preoperative short-course radiotherapy (25 Gy in 5 fractions) or preoperative chemoradiation (50.4 Gy in 28 fractions) with concurrent continuous infusional fluorouracil. The null hypothesis was that chemoradiation resulted in 10% less local recurrences by 3 years (15 vs 5%). After a median follow-up of 5.9 years, the null hypothesis was not rejected. The 3-year local recurrence rate was non-significantly 3.1% greater with the 5-fraction regimen (7.5 vs 4.4%; *p* = 0.24). The 5-year OS was non-significantly better with the 5-fraction regimen (74 vs 70%; *p* = 0.62).

Bujko et al. (46) randomized 541 patients with cT4 or fixed cT3 rectal cancers to either preoperative 25 Gy in 5 fractions with 3 cycles of consolidation FOLFOX4 chemotherapy or preoperative 50.4 Gy in 28 fractions with concurrent oxaliplatin and boluses of 5-fluorouracil and leucovorin. The null hypothesis was that the 5-fraction regimen did not increase the R0 resection rate by 10%. The null hypothesis could not be rejected and the 5-fraction regimen could not be deemed superior because the R0 resection rate was non-significantly improved only by 6% with the 5-fraction regimen (77 vs 71%; *p* = 0.07).

## Discussion

A few generalizations are possible from the above studies. TTFs prolonged the PFS in glioblastoma. In brain metastases, SRS decreased cognitive deterioration compared with WBRT but increased intracranial failures. For head and neck and gynecological cancers, IMRT decreased toxicity, but it remains unclear if it also increased locoregional failures. For early stage NSCLC, a single-fraction SBRT regimen appears very promising. In locally advanced NSCLC, adding durvalumab to radiochemotherapy prolonged PFS, but radiation dose escalation and proton radiotherapy have yet to prove their value. In LD-SCLC, 45 Gy in 30 fractions (given twice a day) remains the standard of care. In ED-SCLC, consolidative radiation therapy did not prove helpful; nor did PCI when there were no brain metastases on the baseline MRI. In breast cancer, IORT could suffice for some patients after lumpectomy and, for those who do require whole breast radiation therapy due to unfavorable pathological features, a hypofractionated regimen is appropriate. In prostate cancer, active monitoring appears to be a reasonable alternative to immediate treatment for clinically localized disease. For those who choose radiation therapy and for those with unfavorable features, there is no proven survival benefit from escalating the radiation dose beyond 70 Gy, but hypofractionated regimens may help decrease treatment burden. Adding ADT to radiotherapy has already prolonged the lives of those with unfavorable features and the addition of abiraterone to ADT appears promising.

### Future Studies

Table 1 summarizes the areas in Radiation Oncology where there is room for improvement for increasing the local control and/or the survival and/or decreasing the adverse effects of treatment and, we hope, may offer a roadmap for future clinical trials of devices and drugs for improving outcomes. Incorporation of tissue, blood, and imaging biomarkers into those trials will help identify subsets of patients most likely to benefit (or be harmed) by the investigational drug or device.

**Table 1:** Current state of the science by anatomic site.

Type of cancer	Trial arms	Null hypothesis	Trial outcomes
Glioblastoma	Surgery, radiation, and chemotherapy with or without tumor-treating fields (TTF) (3)	Adding TTF would not prolong PFS	Median PFS 7.1 (HR = 0.62; *p* = 0.001), median survival 19.6 months (HR = 0.64; *p* = 0.004) Death in 57% at 2 years Gr 3/4 nervous system toxicity 22% Gr 3/4 hematologic toxicity 12% No increase in Gr 3+ toxicity with TTF but increase in mild-to-moderate skin irritation
Anaplastic oligodendroglioma	Surgery and radiation with or without PCV chemotherapy (4)	PCV would not prolong overall survival (OS)	Median survival 4.6 vs 4.7 years Median survival was longer in codeleted tumors treated with PCV (14.7 vs 7.3 years; HR = 0.59; *p* = 0.03) Gr 3/4 toxicity in 65% (most common: hematologic, neurologic, and GI) Fatal chemotherapy induced neutropenia in 1%
	Surgery and radiation with or without PCV chemotherapy (5)	PCV would not prolong OS by 12 months or longer	PCV prolonged median OS by 11.7 months: 42.3 vs 30.6 months; HR = 0.75; *p* = 0.018
Anaplastic glioma, non-codeleted	Surgery followed by 2 × 2 randomization to radiation with or without temozolomide and with or without adjuvant temozolomide (6)	Concurrent or adjuvant temozolomide would not prolong OS	Adjuvant temozolomide improved 5-year survival (55.9% vs 44.1%; HR = 0.65; *p* = 0.0014) Gr 3/4 toxicity in 8–12% with temozolomide
Low-grade glioma	Surgery and radiation with or without PCV chemotherapy (7)	OS would not be improved with PCV	Median survival 13.3 years (HR = 0.59; *p* = 0.003) Death in 28% at 5 years Any grade late events due to radiation in 22%
Brain metastases	Radiosurgery with or without WBRT (8)	Cognitive deterioration at 3 months would not be less after radiosurgery alone	Cognitive deterioration at 3 months improved with radiosurgery: 63.5 vs 91.7% (*p* < 0.001) No difference in survival (10.4 vs 7.4 months)
	Surgery followed by WBRT or stereotactic radiosurgery (9)	OS or cognitive-deterioration-free survival at 6 months would not be superior with radiosurgery	Cognitive deterioration at 6 months was superior with radiosurgery (52 vs 85%; *p* < 0.00031) No difference in survival (12.2 vs 11.6 months)
Head and neck	Intensity-modulated radiation therapy (IMRT) vs 3DCRT (11)	Gr 2 or worse xerostomia at 12 months would not be superior with IMRT	Gr 2 or worse xerostomia in 38% at 12 months with IMRT vs 74% with 3DCRT (*p* = 0.0027)
	Chemoradiotherapy with or without cetuximab (47)	PFS would not be improved with cetuximab	No difference in 3-year PFS with cetuximab (61.2 vs 59.8%) Death in 27.1% at 3 years Local failure in 19.9% at 3 years Metastases in 13% at 3 years Feeding tube dependency at 1 year 21.2%
Lung: non-small cell, early	Stereotactic body radiation therapy (SBRT) in 3 fractions vs conventional radiation in 33 fractions (12)	3-year PFS was not superior with SBRT	3-year PFS 42% in both arms 3-year OS 54% with SBRT
	SBRT with 48 Gy in 4 fractions or 34 Gy in 1 fraction (13)	Each regimen would have an unacceptable Gr 3+ adverse event rate of 17%	Gr 3+ toxicity 10.3–13.3% 2-year survival 61.3 and 77.7% 1-year local control 97.0 and 92.7%
Lung: non-small cell, locally advanced	2 × 2 randomization to standard or high dose chemoradiation with or without cetuximab (15)	OS would not be superior with high-dose radiation or cetuximab	Worse survival with 74 vs 60 Gy (20.3 vs 28.7 months; *p* = 0.008) No benefit to cetuximab Gr 3+ toxicity 76% Gr 3+ pulmonary toxicity 20%
	Chemoradiation with or without adjuvant immunotherapy (durvalumab) (18)	OS or PFS would not be superior with durvalumab	Median PFS 16.8 months with durvalumab (HR = 0.52; *p* < 0.001) No increase in Gr 3/4 toxicity with durvalumab vs placebo (29.9 vs 26.1%)
Lung: small cell, limited stage	Twice-daily vs once-daily chemoradiation (19)	2-year OS would not be superior with once-daily chemoradiation	2-year OS 51 vs 56%; *p* = 0.14 Median survival 30 months (twice-daily) Gr 3+ esophagitis <20%
Lung, small cell, extensive stage	Chemotherapy and prophylactic cranial irradiation (PCI) with or without consolidative thoracic radiotherapy (20)	1-year OS would not be superior with thoracic radiotherapy	1-year OS 33 vs 28%; *p* = 0.066 Median survival 8 months No difference in Gr 3+ toxicity with or without thoracic radiation
	Chemotherapy and PCI with or without consolidative thoracic radiotherapy (21)	OS would not be superior with thoracic radiotherapy	1-year OS 60.1 vs 50.8%; *p* = 0.21 Median survival 15.8 vs 13.8 months Gr 3+ toxicity in 23.8% without and 36.4% with thoracic radiotherapy (*p* = 0.24)
	Chemotherapy with or without (PCI) (22)	OS would not be improved with PCI	Median survival: 11.6 months with PCI vs 13.7 months with observation (HR = 1.27; *p* = 0.094) Less brain metastases with PCI at 6, 12, and 18 months (15.0 vs 46.2%, 32.9 vs 59.0%, 40.1 vs 63.8%; *p* < 0.0001)
Esophagus	Surgery with or without neoadjuvant chemoradiation (33)	OS would not be superior with neoadjuvant chemoradiation	Median survival improved with chemoradiation 49.4 vs 24.0 months; *p* = 0.003 Death in 33% at 2 years Gr 3/4 leukopenia 6% Postoperative morality 4%
Breast: early	Surgery, systemic therapy, and whole breast radiation with or without regional nodal irradiation (RNI) (23)	OS would not be improved with RNI	10-year OS 82.3 vs 80.7%; *p* = 0.06 10-year breast cancer mortality better with RNI 12.5 vs 14.4%; *p* = 0.02 Pulmonary fibrosis with RNI 4.4 vs 1.7% without RNI (*p* < 0.001)
	Surgery, systemic therapy, and whole breast radiation with or without RNI (24)	OS would not be improved with RNI	10-year OS 82.8 vs 81.8%; *p* = 0.38 10-year DFS better with RNI 82.0 vs 77.0%; *p* = 0.01 Gr 2+ pneumonitis 1.2% Gr 2+ lymphedema 8.4%
Pancreas, locally advanced	2 × 2 randomization to chemotherapy with or without erlotinib followed by chemotherapy or chemoradiation (34)	OS would not be improved with erlotinib or radiation	Median survival 15.2 months with and 16.5 months without radiotherapy; *p* = 0.83 No survival improvement with erlotinib Radiotherapy decreased local progression 32 vs 46%; *p* = 0.03 Gr 3/4 hematologic toxicity in 34.1% No increase Gr 3/4 toxicity with chemoradiotherapy except nausea
Prostate: localized PSA detected	Active monitoring, prostatectomy, or radiotherapy (35, 48)	Prostate cancer mortality would not be better with either active monitoring, surgery, or radiotherapy	Prostate cancer-specific death in <2% and no difference between groups (*p* = 0.48) More metastases with active monitoring than surgery or radiation (*p* = 0.004) 6-year use of pads 17% with prostatectomy vs 8% with active-monitoring vs 4% with radiotherapy 6-year adequate erections 17% with prostatectomy vs 30% with active-monitoring vs 27% with radiotherapy
Prostate: intermediate risk	Radiation with or without short course ADT (49)	OS would not be superior with ADT	10-year survival with ADT was 62 vs 57% without; *p* = 0.03 10-year prostate cancer mortality in 4% with ADT vs 8% without; *p* = 0.001 Gr 3+ ADT toxicity <5% Fatal GI toxicity <1%
Prostate: intermediate and high risk	External beam radiation, and ADT, with or without brachytherapy (50, 51)	Biochemical PFS (bPFS) would not be improved with addition of brachytherapy	9-year bPFS was 83% with brachytherapy and 62% without; *p* < 0.001 Death in 22% at 9 years Prostate cancer mortality in 5% at 9 years Gr 3 GU toxicity 18.4% for brachytherapy vs 5.2% without (*p* < 0.001)
Prostate: post-prostatectomy	Salvage radiation with or without ADT (52)	PFS would not be superior with ADT	5-year PFS 80% with ADT vs 62% without; *p* < 0.0001 Gr 2+ ADT-related toxicity 8% Gr 2+ GU toxicity 13%
	Salvage radiation with or without ADT (53)	OS would not be superior with ADT	12-year survival 76.3% with ADT vs 71.3% without; *p* = 0.04 Gynecomastia in 69.7% with ADT vs 10.9% without; *p* < 0.001
Prostate: locally advanced or metastatic	ADT and abiraterone (40)	OS would not be improved with abiraterone	3-year survival, 83% with abiraterone vs 76% without (HR = 0.63; *p* < 0.001) Treatment failure or death in 25% at 3 years (HR = 0.29; *p* < 0.001) Gr 3/4 toxicity 47 vs 33% without abiraterone
Prostate: castration-resistant	Radium-223 or placebo (41)	OS would not be improved with radium-223	Median survival with radium-223 was 14.9 months (HR = 0.70; *p* < 0.001) Gr 3/4 toxicity in 56% Improved quality of life scores with radium-223 Spinal cord compression in 4% One Gr 5 event possibly related to radium-223
Bladder cancer	2 × 2 randomization to radiotherapy with and without chemotherapy followed by whole or partial bladder boost (42, 43)	Locoregional DFS would not be improved with chemotherapy and partial bladder boost would not be non-inferior for 2-year locoregional control	2-year locoregional DFS was 67% with chemotherapy vs 54% without (HR = 0.68; *p* = 0.03) Non-inferiority of partial bladder boost was not established Gr 3/4 toxicity with chemotherapy 36.0 vs 27.5% without; *p* = 0.07 No difference in late Gr 3/4 toxicity with whole or partial bladder boost
Rectal cancer, locally advanced	Neoadjuvant chemoradiation or short course radiation followed by surgery and adjuvant chemotherapy (45)	Local recurrences would be 10% more with short-course radiotherapy	3-year local recurrence was 7.5% with short course vs 4.4% with chemoradiation; *p* = 0.24 5-year survival 74 vs 70%; *p* = 0.62 Late Gr 3/4 toxicity 5.8–8.2%
Anal canal	2 × 2 randomization to radiotherapy with mitomycin or cisplatin with fluorouracil followed by maintenance chemotherapy or not (54)	PFS would not be superior with cisplatin or maintenance chemotherapy	3-year PFS 74% with maintenance chemotherapy and 73% without; *p* = 0.70 3-year PFS without maintenance chemotherapy 73% with mitomycin, and 72% with cisplatin Gr 3/4 toxicity 71% Gr 3/4 skin toxicity 48% Gr 3/4 hematologic toxicity 26%

*Gr, grade; PFS, progression-free survival; DFS, disease-free survival; GI, gastrointestinal; GU, genitourinary; HR, hazard ratio; PCV, procarbazine, lomustine, and vincristine; WBRT, whole brain radiation therapy; 3DCRT, 3D conformal radiation; ADT, androgen deprivation therapy; MMSE, Mini Mental State Examination*.

## Author Contributions

KZ and BV designed the article. All authors (KZ, CNC, and BV) were involved in writing the article, and all authors (KZ, CNC, BV) approved the final manuscript.

## Disclaimer

BV and CNC wrote this in their personal capacity and the opinions expressed herein do not reflect the official position of the NIH and the Department of Health and Human Services.

## Conflict of Interest Statement

The authors declare that the research was conducted in the absence of any commercial or financial relationships that could be construed as a potential conflict of interest.

## References

[1] Vikram B Coleman CN Deye JA. Current status and future potential of advanced technologies in radiation oncology. Part 2. State of the science by anatomic site. Oncology (Williston Park) (2009) 23:380–5. 19476269

[2] Vikram B Coleman CN Deye JA. Current status and future potential of advanced technologies in radiation oncology. Part 1. Challenges and resources. Oncology (Williston Park) (2009) 23:279–83. 19418829

[3] Stupp R Taillibert S Kanner AA Kesari S Steinberg DM Toms SA Maintenance therapy with tumor-treating fields plus temozolomide vs temozolomide alone for glioblastoma: a randomized clinical trial. JAMA (2015) 314:2535–43. 10.1001/jama.2015.16669 26670971

[4] Cairncross G Wang M Shaw E Jenkins R Brachman D Buckner J Phase III trial of chemoradiotherapy for anaplastic oligodendroglioma: long-term results of RTOG 9402. J Clin Oncol (2013) 31:337. 10.1200/JCO.2012.43.2674 23071247 PMC3732012

[5] van den Bent MJ Brandes AA Taphoorn MJ Kros JM Kouwenhoven MC Delattre JY Adjuvant procarbazine, lomustine, and vincristine chemotherapy in newly diagnosed anaplastic oligodendroglioma: long-term follow-up of EORTC brain tumor group study 26951. J Clin Oncol (2013) 31:344–50. 10.1200/JCO.2012.43.2229 23071237

[6] van den Bent MJ Baumert B Erridge SC Vogelbaum MA Nowak AK Sanson M Interim results from the CATNON trial (EORTC study 26053-22054) of treatment with concurrent and adjuvant temozolomide for 1p/19q non-co-deleted anaplastic glioma: a phase 3, randomised, open-label intergroup study. Lancet (2017) 390:1645–53. 10.1016/S0140-6736(17)31442-3 28801186 PMC5806535

[7] Buckner JC Shaw EG Pugh SL Chakravarti A Gilbert MR Barger GR Radiation plus procarbazine, CCNU, and vincristine in low-grade glioma. N Engl J Med (2016) 374:1344–55. 10.1056/NEJMoa1500925 27050206 PMC5170873

[8] Brown PD Jaeckle K Ballman KV Farace E Cerhan JH Anderson SK Effect of radiosurgery alone vs radiosurgery with whole brain radiation therapy on cognitive function in patients with 1 to 3 brain metastases: a randomized clinical trial. JAMA (2016) 316:401–9. 10.1001/jama.2016.9839 27458945 PMC5313044

[9] Brown PD Ballman KV Cerhan JH Anderson SK Carrero XW Whitton AC Postoperative stereotactic radiosurgery compared with whole brain radiotherapy for resected metastatic brain disease (NCCTG N107C/CEC•3): a multicentre, randomised, controlled, phase 3 trial. Lancet Oncol (2017) 18:1049–60. 10.1016/S1470-2045(17)30441-2 28687377 PMC5568757

[10] Patchell RA Tibbs PA Walsh JW A randomized trial of surgery in the treatment of single metastases to the brain. N Engl J Med (1990) 322:494–500. 10.1056/NEJM199002223220802 2405271

[11] Nutting CM Morden JP Harrington KJ Urbano TG Bhide SA Clark C Parotid-sparing intensity modulated versus conventional radiotherapy in head and neck cancer (PARSPORT): a phase 3 multicentre randomised controlled trial. Lancet Oncol (2011) 12:127–36. 10.1016/S1470-2045(10)70290-4 21236730 PMC3033533

[12] Nyman J Hallqvist A Lund JA Brustugun OT Bergman B Bergström P SPACE – a randomized study of SBRT vs conventional fractionated radiotherapy in medically inoperable stage I NSCLC. Radiother Oncol (2016) 121:1–8. 10.1016/j.radonc.2016.08.015 27600155

[13] Videtic GM Hu C Singh AK Chang JY Parker W Olivier KR A randomized phase 2 study comparing 2 stereotactic body radiation therapy schedules for medically inoperable patients with stage I peripheral non-small cell lung cancer: NRG oncology RTOG 0915 (NCCTG N0927). Int J Radiat Oncol Biol Phys (2015) 93:757–64. 10.1016/j.ijrobp.2015.07.2260 26530743 PMC4744654

[14] Chang JY Senan S Paul MA Mehran RJ Louie AV Balter P Stereotactic ablative radiotherapy versus lobectomy for operable stage I non-small-cell lung cancer: a pooled analysis of two randomised trials. Lancet Oncol (2015) 16:630–7. 10.1016/S1470-2045(15)70168-3 25981812 PMC4489408

[15] Bradley JD Paulus R Komaki R Masters G Blumenschein G Schild S Standard-dose versus high-dose conformal radiotherapy with concurrent and consolidation carboplatin plus paclitaxel with or without cetuximab for patients with stage IIIA or IIIB non-small-cell lung cancer (RTOG 0617): a randomised, two-by-two factorial phase 3 study. Lancet Oncol (2015) 16:187–99. 10.1016/S1470-2045(14)71207-0 25601342 PMC4419359

[16] Gore EM Hu C Bar Ad V Robinson CG Wheatley MD Bogart JA Impact of incidental cardiac radiation on cardiopulmonary toxicity and survival for locally advanced non-small cell lung cancer: reanalysis of NRG oncology/RTOG 0617 with centrally contoured cardiac structures. Int J Radiat Oncol Biol Phys (2016) 96:S129–30. 10.1016/j.ijrobp.2016.06.316

[17] Liao Z Lee JJ Komaki R Gomez DR O’Reilly MS Fossella FV Bayesian adaptive randomization trial of passive scattering proton therapy and intensity-modulated photon radiotherapy for locally advanced non-small-cell lung cancer. J Clin Oncol (2018) JCO2017740720. 10.1200/JCO.2017.74.0720 29293386 PMC6008104

[18] Antonia SJ Villegas A Daniel D Vicente D Murakami S Hui R Durvalumab after chemoradiotherapy in stage III non-small-cell lung cancer. N Engl J Med (2017) 377:1919–29. 10.1056/NEJMoa1709937 28885881

[19] Faivre-Finn C Snee M Ashcroft L Appel W Barlesi F Bhatnagar A Concurrent once-daily versus twice-daily chemoradiotherapy in patients with limited-stage small-cell lung cancer (CONVERT): an open-label, phase 3, randomised, superiority trial. Lancet Oncol (2017) 18:1116–25. 10.1016/S1470-2045(17)30318-2 28642008 PMC5555437

[20] Slotman BJ van Tinteren H Praag JO Knegjens JL El Sharouni SY Hatton M Use of thoracic radiotherapy for extensive stage small-cell lung cancer: a phase 3 randomised controlled trial. Lancet (2015) 385:36–42. 10.1016/S0140-6736(14)61085-0 25230595

[21] Gore EM Hu C Sun AY Grimm DF Ramalingam SS Dunlap NE Randomized phase II study comparing prophylactic cranial irradiation alone to prophylactic cranial irradiation and consolidative extracranial irradiation for extensive disease small cell lung cancer (ED-SCLC): NRG oncology RTOG 0937. J Thorac Oncol (2017) 12:1561–70. 10.1016/j.jtho.2017.06.015 28648948 PMC5610652

[22] Takahashi T Yamanaka T Seto T Harada H Nokihara H Saka H Prophylactic cranial irradiation versus observation in patients with extensive-disease small-cell lung cancer: a multicentre, randomised, open-label, phase 3 trial. Lancet Oncol (2017) 18:663–71. 10.1016/S1470-2045(17)30230-9 28343976

[23] Poortmans PM Collette S Kirkove C Van Limbergen E Budach V Struikmans H Internal mammary and medial supraclavicular irradiation in breast cancer. N Engl J Med (2015) 373:317–27. 10.1056/NEJMoa1415369 26200978

[24] Whelan TJ Olivotto IA Parulekar WR Ackerman I Chua BH Nabid A Regional nodal irradiation in early-stage breast cancer. N Engl J Med (2015) 373:307–16. 10.1056/NEJMoa1415340 26200977 PMC4556358

[25] Vaidya JS Wenz F Bulsara M Tobias JS Joseph DJ Keshtgar M Risk-adapted targeted intraoperative radiotherapy versus whole-breast radiotherapy for breast cancer: 5-year results for local control and overall survival from the TARGIT-A randomised trial. Lancet (2014) 383:603–13. 10.1016/S0140-6736(13)61950-9 24224997

[26] Cuzick J Radiotherapy for breast cancer, the TARGIT-A trial. Lancet (2014) 383:1716 10.1016/S0140-6736(14)60826-6 24835608

[27] Veronesi U Orecchia R Maisonneuve P Viale G Rotmensz N Sangalli C Intraoperative radiotherapy versus external radiotherapy for early breast cancer (ELIOT): a randomised controlled equivalence trial. Lancet Oncol (2013) 14:1269–77. 10.1016/S1470-2045(13)70497-2 24225155

[28] Strnad V Ott OJ Hildebrandt G Kauer-Dorner D Knauerhase H Major T 5-year results of accelerated partial breast irradiation using sole interstitial multicatheter brachytherapy versus whole-breast irradiation with boost after breast-conserving surgery for low-risk invasive and in-situ carcinoma of the female breast: a randomised, phase 3, non-inferiority trial. Lancet (2016) 387:229–38. 10.1016/S0140-6736(15)00471-7 26494415

[29] START Trialists’ Group Bentzen SM Agrawal RK Aird EG Barrett JM Barrett-Lee PJ The UK Standardisation of Breast Radiotherapy (START) trial A of radiotherapy hypofractionation for treatment of early breast cancer: a randomised trial. Lancet Oncol (2008) 9:331–41. 10.1016/S1470-2045(08)70077-9 18356109 PMC2323709

[30] START Trialists’ Group Bentzen SM Agrawal RK Aird EG Barrett JM Barrett-Lee PJ The UK Standardisation of Breast Radiotherapy (START) trial B of radiotherapy hypofractionation for treatment of early breast cancer: a randomised trial. Lancet (2008) 371:1098–107. 10.1016/S0140-6736(08)60348-7 18355913 PMC2277488

[31] Haviland JS Owen JR Dewar JA Agrawal RK Barrett J Barrett-Lee PJ The UK Standardisation of Breast Radiotherapy (START) trials of radiotherapy hypofractionation for treatment of early breast cancer: 10-year follow-up results of two randomised controlled trials. Lancet Oncol (2013) 14:1086–94. 10.1016/S1470-2045(13)70386-3 24055415

[32] Whelan TJ Pignol JP Levine MN Julian JA MacKenzie R Parpia S Long-term results of hypofractionated radiation therapy for breast cancer. N Engl J Med (2010) 362:513–20. 10.1056/NEJMoa0906260 20147717

[33] van Hagen P Hulshof MC van Lanschot JJ Steyerberg EW van Berge Henegouwen MI Wijnhoven BP Preoperative chemoradiotherapy for esophageal or junctional cancer. N Engl J Med (2012) 366:2074–84. 10.1056/NEJMoa1112088 22646630

[34] Hammel P Huguet F van Laethem JL Goldstein D Glimelius B Artru P Effect of chemoradiotherapy vs chemotherapy on survival in patients with locally advanced pancreatic cancer controlled after 4 months of gemcitabine with or without erlotinib: the LAP07 randomized clinical trial. JAMA (2016) 315:1844–53. 10.1001/jama.2016.4324 27139057

[35] Hamdy FC Donovan JL Lane JA Mason M Metcalfe C Holding P 10-year outcomes after monitoring, surgery, or radiotherapy for localized prostate cancer. N Engl J Med (2016) 375:1415–24. 10.1056/NEJMoa1606220 27626136

[36] Michalski JM Moughan J Purdy J Bosch W Bruner DW Bahary JP Effect of standard vs dose-escalated radiation therapy for patients with intermediate-risk prostate cancer: the NRG oncology RTOG 0126 randomized clinical trial. JAMA Oncol (2018). 10.1001/jamaoncol.2018.0039 29543933 PMC5885160

[37] Dearnaley D Syndikus I Mossop H Khoo V Birtle A Bloomfield D Conventional versus hypofractionated high-dose intensity-modulated radiotherapy for prostate cancer: 5-year outcomes of the randomised, non-inferiority, phase 3 CHHiP trial. Lancet Oncol (2016) 17:1047–60. 10.1016/S1470-2045(16)30102-4 27339115 PMC4961874

[38] Lee WR Dignam JJ Amin MB Bruner DW Low D Swanson GP Randomized phase III noninferiority study comparing two radiotherapy fractionation schedules in patients with low-risk prostate cancer. J Clin Oncol (2016) 34:2325–32. 10.1200/JCO.2016.67.0448 27044935 PMC4981980

[39] Catton CN Lukka H Gu CS Martin JM Supiot S Chung PWM Randomized trial of a hypofractionated radiation regimen for the treatment of localized prostate cancer. J Clin Oncol (2017) 35:1884–90. 10.1200/JCO.2016.71.7397 28296582

[40] James ND de Bono JS Spears MR Clarke NW Mason MD Dearnaley DP Abiraterone for prostate cancer not previously treated with hormone therapy. N Engl J Med (2017) 377:338–51. 10.1056/NEJMoa1702900 28578639 PMC5533216

[41] Parker C Nilsson S Heinrich D Helle SI O’Sullivan JM Fosså SD Alpha emitter radium-223 and survival in metastatic prostate cancer. N Engl J Med (2013) 369:213–23. 10.1056/NEJMoa1213755 23863050

[42] James ND Hussain SA Hall E Jenkins P Tremlett J Rawlings C Radiotherapy with or without chemotherapy in muscle-invasive bladder cancer. N Engl J Med (2012) 366:1477–88. 10.1056/NEJMoa1106106 22512481

[43] Huddart RA Hall E Hussain SA Jenkins P Rawlings C Tremlett J Randomized noninferiority trial of reduced high-dose volume versus standard volume radiation therapy for muscle-invasive bladder cancer: results of the BC2001 trial (CRUK/01/004). Int J Radiat Oncol Biol Phys (2013) 87:261–9. 10.1016/j.ijrobp.2013.06.2044 23958147 PMC3753507

[44] Klopp AH Yeung AR Deshmukh S A phase III randomized trial comparing patient-reported toxicity and quality of life (QOL) during pelvic intensity modulated radiation therapy as compared to conventional radiation therapy. Int J Radiat Oncol Biol Phys (2016) 96(2):S3 10.1016/j.ijrobp.2016.06.024

[45] Ngan SY Burmeister B Fisher RJ Solomon M Goldstein D Joseph D Randomized trial of short-course radiotherapy versus long-course chemoradiation comparing rates of local recurrence in patients with T3 rectal cancer: Trans-Tasman Radiation Oncology Group trial 01.04. J Clin Oncol (2012) 30:3827–33. 10.1200/JCO.2012.42.9597 23008301

[46] Bujko K Wyrwicz L Rutkowski A Malinowska M Pietrzak L Kryński J Long-course oxaliplatin-based preoperative chemoradiation versus 5 × 5 Gy and consolidation chemotherapy for cT4 or fixed cT3 rectal cancer: results of a randomized phase III study. Ann Oncol (2016) 27:834–42. 10.1093/annonc/mdw062 26884592

[47] Ang KK Zhang Q Rosenthal DI Nguyen-Tan PF Sherman EJ Weber RS Randomized phase III trial of concurrent accelerated radiation plus cisplatin with or without cetuximab for stage III to IV head and neck carcinoma: RTOG 0522. J Clin Oncol (2014) 32:2940–50. 10.1200/JCO.2013.53.5633 25154822 PMC4162493

[48] Donovan JL Hamdy FC Lane JA Mason M Metcalfe C Walsh E Patient-reported outcomes after monitoring, surgery, or radiotherapy for prostate cancer. N Engl J Med (2016) 375:1425–37. 10.1056/NEJMoa1606221 27626365 PMC5134995

[49] Jones CU Hunt D McGowan DG Amin MB Chetner MP Bruner DW Radiotherapy and short-term androgen deprivation for localized prostate cancer. N Engl J Med (2011) 365:107–18. 10.1056/NEJMoa1012348 21751904

[50] Morris WJ Tyldesley S Rodda S Halperin R Pai H McKenzie M Androgen suppression combined with elective nodal and dose escalated radiation therapy (the ASCENDE-RT Trial): an analysis of survival endpoints for a randomized trial comparing a low-dose-rate brachytherapy boost to a dose-escalated external beam boost for high- and intermediate-risk prostate cancer. Int J Radiat Oncol Biol Phys (2017) 98:275–85. 10.1016/j.ijrobp.2016 28262473

[51] Rodda S Tyldesley S Morris WJ Keyes M Halperin R Pai H ASCENDE-RT: an analysis of treatment-related morbidity for a randomized trial comparing a low-dose-rate brachytherapy boost with a dose-escalated external beam boost for high- and intermediate-risk prostate cancer. Int J Radiat Oncol Biol Phys (2017) 98:286–95. 10.1016/j.ijrobp.2017.01.008 28433432

[52] Carrie C Hasbini A de Laroche G Richaud P Guerif S Latorzeff I Salvage radiotherapy with or without short-term hormone therapy for rising prostate-specific antigen concentration after radical prostatectomy (GETUG-AFU 16): a randomised, multicentre, open-label phase 3 trial. Lancet Oncol (2016) 17:747–56. 10.1016/S1470-2045(16)00111-X 27160475

[53] Shipley WU Seiferheld W Lukka HR Major PP Heney NM Grignon DJ. Radiation with or without antiandrogen therapy in recurrent prostate cancer. N Engl J Med (2017) 376:417–28. 10.1056/NEJMoa1607529 28146658 PMC5444881

[54] James RD Glynne-Jones R Meadows HM Cunningham D Myint AS Saunders MP Mitomycin or cisplatin chemoradiation with or without maintenance chemotherapy for treatment of squamous-cell carcinoma of the anus (ACT II): a randomised, phase 3, open-label, 2 × 2 factorial trial. Lancet Oncol (2013) 14:516–24. 10.1016/S1470-2045(13)70086-X 23578724

